# Oxidative Stress and Lipid Peroxidation: Prospective Associations Between Ferroptosis and Delayed Wound Healing in Diabetic Ulcers

**DOI:** 10.3389/fcell.2022.898657

**Published:** 2022-07-08

**Authors:** Jiawei Feng, Jialin Wang, Yuqing Wang, Xiaoting Huang, Tengteng Shao, Xiaofei Deng, Yemin Cao, Mingmei Zhou, Cheng Zhao

**Affiliations:** ^1^ Shanghai Traditional Chinese Medicine Integrated Hospital, Shanghai University of Traditional Chinese Medicine, Shanghai, China; ^2^ Graduate School, Shanghai University of Traditional Chinese Medicine, Shanghai, China; ^3^ Institute for Interdisciplinary Medicine Sciences, Shanghai University of Traditional Chinese Medicine, Shanghai, China

**Keywords:** diabetic ulcer, ferroptosis, oxidative stress, lipid peroxidation, delayed wound healing

## Abstract

Diabetic ulcers are one of the major complications of diabetes, and patients usually suffer from amputation and death due to delayed ulcer wound healing. Persistent inflammation and oxidative stress at the wound site are the main manifestations of delayed wound healing in diabetic ulcers. In addition, chronic hyperglycemia in patients can lead to circulatory accumulation of lipid peroxidation products and impaired iron metabolism pathways leading to the presence of multiple free irons in plasma. Ferroptosis, a newly discovered form of cell death, is characterized by intracellular iron overload and accumulation of iron-dependent lipid peroxides. These indicate that ferroptosis is one of the potential mechanisms of delayed wound healing in diabetic ulcers and will hopefully be a novel therapeutic target for delayed wound healing in diabetic patients. This review explored the pathogenesis of diabetic ulcer wound healing, reveals that oxidative stress and lipid peroxidation are common pathological mechanisms of ferroptosis and delayed wound healing in diabetic ulcers. Based on strong evidence, it is speculated that ferroptosis and diabetic ulcers are closely related, and have value of in-depth research. We attempted to clarify prospective associations between ferroptosis and diabetic ulcers in terms of GPX4, iron overload, ferroptosis inhibitors, AGEs, and HO-1, to provide new ideas for exploring the clinical treatment of diabetic ulcers.

## 1 Introduction

Diabetic ulcers, a major complication of diabetes, mostly appears in the legs and feet of diabetic patients (Everett and Mathioudakis, 2018). At present, the number of diabetic patients with ulcers accounts for about 20% of the total number of diabetic patients. About 50%–70% of limb amputations are caused by delayed wound healing of persistent diabetic ulcers. According to reports, every 30 s in the world, one diabetic patient suffers from amputation due to persistent diabetic chronic ulcers, and the annual cost of medical costs and lots of productivity due to diabetic wounds is more than 200 billion US dollars (Al-Mohaithef et al., 2022). Despite recent advances in the understanding of wound healing, much still remains unknown about the molecular mechanisms underlying impaired wound healing in diabetes, and the efficacy shown by existing approaches is unsatisfactory (Everett and Mathioudakis, 2018). The glucose metabolism capacity decreases in diabetic patients, which causes the ulcer wound to be in a state of hyperglycemia, and further leads to impaired angiogenesis and delayed wound healing in diabetes (Hajmousa et al., 2018). Long-term hyperglycemia in diabetics causes increased mitochondrial reactive oxygen species (ROS) production, leading to the activation of lipid peroxidation, which, unless cleared by the cellular antioxidant system, can lead to cellular dysfunction and death by destroying macromolecules (Cheng et al., 2018; Icli et al., 2019).

First defined in 2012, ferroptosis is a form of cell death described as a non-apoptotic peroxidation-induced cell death that is dependent on the availability of ROS and iron (Hajmousa et al., 2018). As it takes place without caspases, a family of cysteine proteases cleaving specific intracellular substrates leading to apoptosis (Singh et al., 2013; Everett and Mathioudakis, 2018; Dolp et al., 2019), ferroptosis is characterized by the overload of intracellular iron and the accumulation of iron-dependent lipid peroxide. Additionally, ferroptosis also causes the inhibition of oxidoreductases, particularly glutathione peroxidase 4 (GPX4), a lipid peroxide scavenger (Icli et al., 2019). In terms of biochemistry, iron metabolism, nicotinamide adenine dinucleotide phosphate (NADPH) oxidase activity, and accumulation of ROS in lipid peroxidation products collectively constitute the major features of ferroptosis. Therefore, ferroptosis might modulate the pathological role of ROS and lipid peroxidation products, which impair wound healing in diabetics.

**FIGURE 1:**
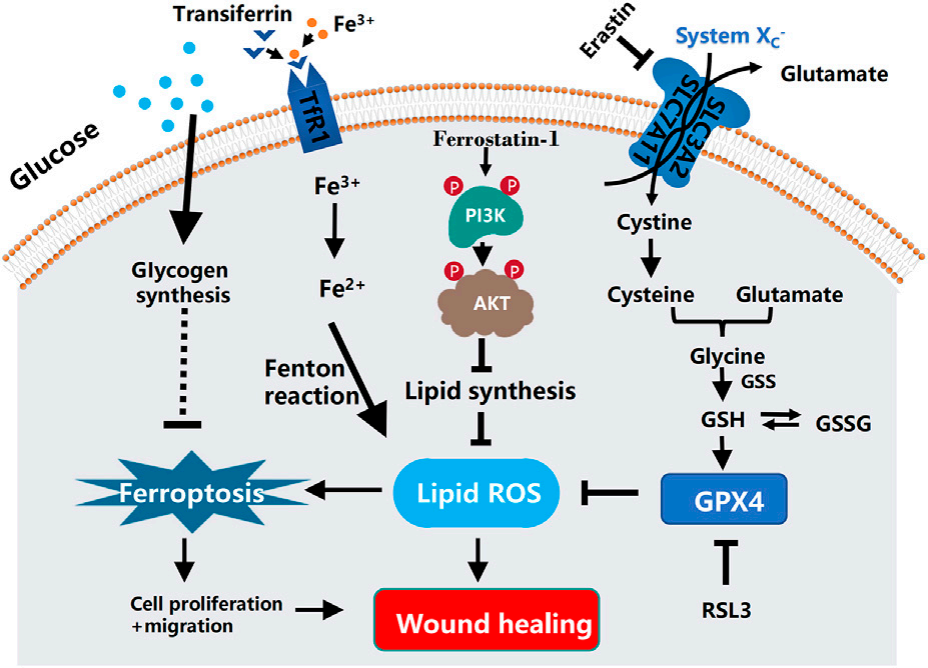
Schematic diagram of the mechanism of ferroptosis in diabetic ulcers and the mechanism of ferrostatin-1 repairing diabetic ulcers by inhibiting ferroptosis through PI3K-AKT. In diabetic ulcers, the synthesis of lipid peroxidation products is gradually increased as the increasing amount of glucose enters the cells. Under the action of intracellular iron ions, lipid peroxides continue to accumulate, thereby attacking cellular DNA and other biological macromolecules in the granulation tissue of ulcerated skin, ultimately triggering cellular ferroptosis. Ferrostatin-1, an inhibitor of ferroptosis, can reduce lipid peroxidative damage by activating the PI3K-AKT signaling pathway to block the pathway of ferroptosis, and at the same time promoting the regeneration, differentiation, and migration of vascular endothelial cells and epithelial cells, thereby effectively improving the ability of wound healing. In the process of ferroptosis, SLC7A11, SLC3A2, and the consume of glutathione lead to the accumulation of iron-dependent ROS. In addition, glucose and glutamine metabolism will decrease the levels of glutathione and glutathione peroxidase 4 (GPX4), resulting in the production of iron-independent reactive oxygen species. Ferroptosis is mediated by excess iron between cells. Ferric iron (Fe^3+^) binds to transferrin and is imported into cells through the membrane protein transferrin receptor 1 (TfR1). Then, Fe3^+^ is reduced to Fe2+ by reductase. Finally, excess intracellular iron generates ROS through the Fenton reaction, which triggers ferroptosis.

Long-lasting hyperglycemia in diabetic patients leads to the impairment of iron metabolic pathway, a significant reduction in the availability of iron-biding sites in circulating transferrin (Tf) and ferrin (SF), and the presence of multiple free irons in plasma. Impaired insulin signaling in diabetic patients results in insufficient hepcidin synthesis, increased intestine iron apparent absorption, and elevated circulating iron level. Increased free iron in plasma leads to oxidative stress and ferroptosis (Alavi et al., 2014; Jiang et al., 2019). Hence, therapies aimed to modulate iron metabolism are critical for developing effective prevention and treatment for type 2 diabetes mellitus (T2DM) and its related disorders. The crucial involvement of ferroptosis in cell growth and survival, as well as the presence of redox imbalances in diabetic wounds, has made ferroptosis a promising therapeutic target for delayed wound healing in diabetic patients. A schematic diagram of the mechanism of ferroptosis in diabetic ulcers is shown in Figure 1. In this prospective review, we firstly describe the pathological basis associated with refractory wounds in diabetic ulcers, such as inflammation, hypoxia, and oxidative stress. Next, we elaborate the pathological mechanisms and risk factors of ferroptosis, with an emphasis on the relationship between lipid peroxidation and oxidative stress. In addition, we attempted to explore its relationship with oxidative stress and lipid peroxidation from ferroptosis inducers and inhibitors to further explore the pathological mechanism of ferroptosis and delayed wound healing in diabetes. Finally, based on the common mechanisms of GPX4, iron overload, ferroptosis inhibitors, AGEs, and HO-1, it is further predicted that ferroptosis is an important link in the pathological mechanisms of delayed healing of diabetic ulcer wounds. The ferroptosis research will provide more possibilities for the clinical treatment of diabetic ulcers and the development of new drugs in the future.

## 2 Pathogenesis of Delayed Wound Healing in Diabetic Ulcers

Wound healing is a multifactorial and dynamic process, and its treatment goal is to restore the integrity of anatomical structure and function. The primary requirement of wound treatment is to achieve healed quickly and completely without diffuse infection and sepsis. According to the situation of wound healing, ulcer wound healing can be divided into chronic and acute. Acute wounds heal as expected and undergo a normally, orderly and timely repair process, thereby continuing to restore the integrity of anatomical and functional (Singh et al., 2013). The healing process can involve various cells and is divided into five overlapping stages, including hemostasis, inflammation, proliferation, contraction and remodeling.

The acute wound healing process starts with hemostasis to prevent blood loss and microbial invasion of the wound, and then enters the inflammation stage. At this stage, pro-inflammatory cells and neutrophils are upregulated, and pathogens, debris, growth factors and other cytokines are eliminated by macrophages. The proliferative phase overlaps with the inflammation phase. In the inflammatory phase, the damaged area is activated and filled with new tissues, new blood vessels and matrix structures. In the final remodeling phase, the extracellular matrix’s tensile strength increases and the blood supply in the damage area decreases (Singh et al., 2013; Dolp et al., 2019). Chronic trauma can be defined as continuous intervention on any tissue, but it takes a long time to heal or even non-healing or recurrence (Singh et al., 2013). Many factors, such as chronic diseases, vascular insufficiency (Zhao et al., 2016), diabetes, malnutrition, aging, and other local factors such as stress, infection and edema (Zhao et al., 2016), can lead to delayed wound healing. Diabetes-related peripheral neuropathy coupled with interruption of perfusion can easily lead to poor structure of the foot, loss of consciousness, and increase the risk of ulcers due to repeated mechanical stress. Moreover, patients with diabetic ulcers are prone to complications, including functional limitations, difficulty walking, and infections such as cellulitis, abscesses, osteomyelitis, gangrene, and sepsis. These recurring complications aggravate the difficulty of healing, resulting in the delayed wound healing. The pathological mechanisms of ulcer healing in diabetic patients have not been clearly defined. Here we review the related factors of wound healing disorder in diabetic ulcers from the following aspects (Table 1).

**TABLE 1:** Pathogenesis of delayed wound healing of diabetic ulcers.

Influencing factor	Target	Conventional role
Increased inflammatory cells and proinflammatory factors	Macrophage inflammatory protein factor	Expressed in the form of membrane binding on the surface of inflammatory cells, epithelial cells, macrophages, and vascular smooth muscle cells
	Macrophage polarization	Caused by hyperglycemia and oxidative stress and be the major reason for delayed wound healing
Insufficient oxygen supply to ulcer wound	Narrowing of blood vessels	Causes insufficient oxygen supply to the wound
	Glycosylation of haemoglobin	Causes insufficient supply of nutrients and oxygen in the tissues, thereby delays the healing process
	Metamorphin skin tissue damage	Activated after injury and induces increased expression of pro-inflammatory chemokines and aggravates the cellular stress response by promoting the accumulation of unfolded proteins in the endoplasmic reticulum
Ulcer wound ischemia	MiR-210	Plays a crucial role in limiting the proliferation of keratinocytes and delays wound healing
	MiR-200b	Derepresses the transcription factor endothelial transcription factor GATA2 and VEGFR2 to turn on wound angiogenesis
	MiR-21, miR-198, miR-130a, miR-26A, and miR-146	Involved in re-epithelialization, delayed inflammatory response, fibroblast migration, keratinocyte migration and angiogenesis in the diabetic wounds
	Stromal cell proteins	Bind to a variety of proteins in ECM library and connect to homologous cell surface receptors
	Angiopoietin-like factor 4 (AL-4)	Delays the healing process by influencing angiogenesis and re-epithelialization
Diabetic peripheral neuropathy	Abnormal glycosylation of neuronal proteins and abnormal activation of protein kinase C	Lead to neurological dysfunction and ischemia under conditions of hyperglycemia and oxidative stress
Growth factors	VEGF	Increases blood capillary density and improves blood perfusion and metabolism in injured tissues
	IGF-1, IGF-2, TGF-β, PDGF, EGF, TNF-α, and IL-6	Play a key role in initiating and maintaining different stages of wound healing
Oxidative stress	NRF2	Reduces apoptosis and promotes cell migration, proliferation and cell differentiability by regulating the adaptive response to oxidative stress
	ATF-3	Induces lymphatic B cell dysregulation and promote the occurrence of diabetic complications

### 2.1 Changes in Inflammatory Cells, Proinflammatory Factors and Growth Factors

#### 2.1.1 Increased Inflammatory Cells and Proinflammatory Factors

Generally, wound healing is a process mediated by growth factors and cytokines released by different cells (Davis et al., 2018) such as fibroblasts, endothelial cells, phagocytes, platelets, and keratinocytes (Davis et al., 2018) activated by the immune response after the skin barrier is broken. The generation and adjustment of various growth factors and cytokines are ardently engaged by these cells. Under the action of inflammatory chemical attractants, monocytes are activated to become macrophages, and then more growth factors are secreted, including platelet-derived growth factor (PDGF) and vascular endothelial growth factor (VEGF) (Dixon and Edmonds, 2021). Neutrophils and lymphoid T and B cells produce tumor necrosis factor alpha (TNF-α) and interleukin-10 (IL-10). Fibroblasts, keratinocytes, endothelial cells and mast cells are also involved in the production of insulin-like growth factor-1 (IGF-1), transforming growth factor-beta (TGF-β) and VEGF. Proinflammatory cytokines such as macrophage inflammatory protein factor, keratinocyte chemokine and β-Defensin play an essential role in leukocyte aggregation (Brem and Tomic-Canic, 2007; Hajmousa et al., 2018; Icli et al., 2019). Badr, 2013 have proved that macrophage inflammatory protein factor in the blood of diabetic patients is expressed in the form of membrane binding on the surface of inflammatory cells, epithelial cells, macrophages and vascular smooth muscle cells. However, in diabetic ulcer wounds, the expression of cellular inflammatory protein factors increased, the level of β-Defensin decreased, the sensor activation signal and transcription activator were abnormally activated, and the activity of nuclear factor kappa B (NF-kappaB) and protein kinase B (PKB) decreased. All the above factors collectively contribute to the delayed healing of diabetic wounds (Brem and Tomic-Canic, 2007; Hajmousa et al., 2018; Icli et al., 2019). Furthermore, macrophage polarization, caused by hyperglycemia and oxidative stress, is the major reason for delayed wound healing (Basu Mallik et al., 2018; Jiang et al., 2019). Research has found that there are many phenomena in diabetic animal ulcer models, including the continuous generation of pro-inflammatory cytokines, damaged functions of macrophages and neutrophils, injured migration and proliferation of keratinocytes and fibroblasts and reduced production of healing-related factors (such as growth factors) (Alavi et al., 2014; Okizaki et al., 2015).

#### 2.1.2 Defects in the Number and Type of Growth Factors

Wound healing is a complex physiological process involving (Park et al., 2017) the interaction between various types of cells, growth factors, extracellular matrix (ECM) components and proteases. Growth factors, as biologically active peptides, are mainly involved in the early inflammation stage of the granulation stage of tissue formation. Due to changes in growth factor expression, reduction in production, reduction in release, capture and excessive degradation (Crovetti et al., 2004), the number and types of growth factors in wounds are often defective. In addition, the stabilization between matrix formation and degradation is one of the main characteristics of wound healing. In diabetic patients, vascular endothelial growth factor (VEGF) (Biswas et al., 2010; Kim et al., 2018), insulin-like growth factor 1 (IGF-1), insulin-like growth factor 2 (IGF-2) (Capilla-Gonzalez et al., 2018; Zeng et al., 2019), transforming growth factor beta (TGF-β) (Li et al., 2019), platelet-derived growth factor (PDGF) (Zahid et al., 2019), epidermal growth factor (EGF) (Jee et al., 2019), TNF-α and interleukin 6 (IL-6) were significantly reduced. Growth factors play a crucial role in initiating and maintaining different stages of wound healing. The wound healing in diabetic patients may be delayed by the downregulation of growth factor receptors and the brisk degradation of growth factors. VEGF is one of the most valuable angiogenesis cytokines during wound healing. The content of VEGF in wounds can significantly affect healing and support angiogenesis (Melincovici et al., 2018). VEGF increases blood capillary density and improves blood perfusion and metabolism in damage areas. Restoration of blood flow of ulcer skin tissue is conducive to the supply of nutrients and oxygen to participate in the forming of granulation tissue, help the growth of repairing cells and promote wound healing. It can be seen that VEGF is the main regulator of wound revascularization. The role of VEGF depends on the activation of its receptor. The activation of VEGFR-1 leads to inflammation, while the activation of VEGFR-2 leads to angiogenesis (Farghaly et al., 2021). It was found that the abnormal pattern of VEGF receptor, the decrease of VEGF’s mRNA level, the increase of VEGFR-1 level and the decrease of VEGFR-2 level are the essential reasons for the failure of wound healing (Zhou et al., 2015).

### 2.2 Diabetic Peripheral Neuropathy and Insufficient Oxygen Supply to the Wound

Diabetic peripheral neuropathy can cause disorder of motor, sensory and autonomic nerve function and it also can cause delayed wound healing. Loss of pain, a sensory neuropathy, is a major threat to the regeneration of diabetic ulcer wound. Under conditions of hyperglycemia and oxidative stress, abnormal glycosylation of neuronal proteins and abnormal activation of protein kinase C can lead to neurological dysfunction and ischemia. Patients with diabetic ulcers will worsen their wounds due to their lack of awareness of the wound (Huang et al., 2015). The healing process of ulcers in diabetic patients is also stagnant due to other factors. These factors include metabolic defects, hypoxia caused by hemoglobin glycation, changes in red blood cell membrane permeability (Brem and Tomic-Canic, 2007), and narrowing of blood vessels. Diabetic ulcer wounds have insufficient oxygen supply to the wound site because of narrowing of blood vessels. The glycosylation of hemoglobin further leads to insufficient supply of nutrients and oxygen in the tissues, thereby delaying the healing process. In addition, metamorphin skin tissue is activated immediately after injury. By promoting the accumulation of unfolded proteins in the endoplasmic reticulum, it induces increased expression of pro-inflammatory chemokines and aggravates the cellular stress response (Schurmann et al., 2014).

### 2.3 Ischemia of Ulcer Wound

The local ischemia caused by diabetic microvascular complications has greatly hindered wound healing. Micro RNA (miRNA or miR), a small non-coding RNA molecule with a length of 18–25 nucleotides, plays an important role in diabetic microvascular complications. Changes in tight miRNA regulation may cause serious physiological abnormalities such as diabetes and other chronic diseases and their complications (Moura et al., 2014). *In vivo* imaging revealed that massive hypoxia inducible factor HIF-1α is stabilized in ischemic wounds, whereas HIF-1α induced microRNA-210(miR-210)expression. MiR-210 plays a key role in limiting the proliferation of keratinocytes (Narayanan et al., 2020) and delaying wound healing. Downregulation of miR-200b depressed the transcription factor endothelial transcription factor GATA binding protein 2 (GATA2) and vascular endothelial growth factor receptor 2 (VEGFR2) (Chan et al., 2012) to turn on wound angiogenesis, which is destroyed in diabetic wounds. In addition, other subtypes of miRNA, such as miR-21, miR-198, miR-130a, miR-26A, and miR-146, are involved in re-epithelialization, delayed inflammatory response, fibroblast migration, keratinocyte migration and angiogenesis in the diabetic wounds (Bhattacharya et al., 2016; Icli et al., 2016). Other factors include increased serum matrix metalloproteinases (Li et al., 2013), decreased collagen type ratio, dysregulated expression of skin neuropeptides (Nowak et al., 2021), lack of thrombin activated fibrinolytic inhibitor (Bryk-Wiązania and Undas, 2021), reduced platelet growth factor and modification of late phase three advanced glycation end products (Nass et al., 2010), decreased number of epidermal nerves (Alavi et al., 2014), and the increase of extracellular matrix components and the matrix metalloproteinase reconstitution are imbalanced (Alavi et al., 2014). Studies have announced the role of stromal cell proteins in wound healing. Stromal cell (Chong et al., 2014) proteins can bind to a variety of proteins in the extracellular matrix (ECM) library and connect to homologous cell surface receptors.

Angiopoietin-like factor 4 (ANGPTL4) is a stromal cell protein that plays an important role in lipid and glucose metabolism and promotes keratinocyte migration, proliferation and angiogenesis. Blood glucose concentrations were inversely correlated with ANGPTL4, and in patients with type 2 diabetes, their blood glucose concentrations were higher, but ANGPTL4 concentrations were lower (Arya et al., 2014). ANGPTL4 initiates the activation of the Janus kinase-signal transducer and activator of transcription 3 (JAK/STAT3), mediated upregulation of inducible nitric oxide synthase (iNOS) expression in wound epithelium, and promotes angiogenesis. In a typical wound injury, the expression of ANGPTL4 is significantly increased. On the contrary, the expression of ANGPTL4 is still very low during the entire healing process of diabetic wounds (Chong et al., 2014). This indicates that by influencing angiogenesis and re-epithelialization, ANGPTL4 delays the healing process.

### 2.4 Oxidative Stress in Wound Healing of Diabetic Ulcers

Molecular mechanisms affecting the healing of diabetic ulcers are continuously being discovered, and the diacylglycerol pathway, hexose pathway, nitric oxide blocking pathway, polyol pathway, protein kinase C pathway, and advanced glycation end products (AGEs) have been recognized. These mechanisms are caused by excessive production of ROS by mitochondria (Bhattacharya et al., 2016) and oxidative stress stimulation. During the development of diabetes, extreme oxidative stress plays an essential role in the complications of diabetes, such as ulcer healing disorders. Among them, the nuclear factor erythroid 2-related factor 2 (NRF2) reduces apoptosis, promotes cell migration, proliferation and cell differentiability by regulating the adaptive response to oxidative stress (Yu and Xiao, 2021). NRF2 is activated by oxidative stress and high glucose to regulate and repair damage. Long et al. (2016) have proven that the excessive oxidative stress levels caused by diabetes can be reduced by inducing activation of NRF2 to regulate matrix metalloproteinase 9 (MMP-9), TGF-β and gene expression associated with migration and proliferation. Activating transcription factor 3 (ATF-3) is a stress-inducing gene whose expression can induce lymphatic B cell dysregulation and promote the occurrence of diabetic complications (Zhou et al., 2018). Excessive pro-inflammatory response activates ATF-3 and inducible nitric oxide synthase (iNOS), which leads to the occurrence of oxidative stress and becomes another cause of prolonged wound healing. Badr et al. reported the upregulated expression level of ATF-3 and iNOS accompanied by increasing free radicals and activities of caspase-3, -8, and -9. One of the important reasons for the damage of cell differentiation and remodeling during the healing process (Badr et al., 2016).

### 2.5 Lipid Peroxidation and Diabetes

Oxidative stress occurs when the balance between reactive oxygen species (ROS) and antioxidant defense is disturbed, resulting in tissue damage (Jelic et al., 2021). Sources of oxidative stress in diabetes include ROS produced by auto-oxidation reactions of sugars and sugar adducts to protein and by autoxidation of unsaturated lipids in plasma and membrane protein (Dham et al., 2021). ROS attack a variety of substances, of which lipids leading to lipid peroxidation are the main target compounds. Lipid peroxidation occurs when oxidants like ROS attack lipids containing carbon-carbon double bonds, especially the process of polyunsaturated fatty acids (PUFAs) (Ayala et al., 2014). There are two main categories of lipid peroxidation products: hydroperoxides and reactive carbonyl species (RCS). RCS impairs various cellular processes such as energy production (Humphries et al., 1998) and ion channel activity (Bhatnagar, 1995), etc. Moreover, RCS can also regulate cell proliferation, stress adaptation and signaling (Singh et al., 2015). RCS levels can be used as a biomarker to identify the severity of diabetic complications, as RCS levels correlate with the severity of organ damage in diabetic patients (Dham et al., 2021). 4-Hydroxy-2-nonenal (4-HNE) is one of the most biologically active and well-studied cell-reactive aldehydes (Dham et al., 2021). 4-HNE is generally considered to be a marker of oxidative stress (Leake et al., 2012), so the level of 4-HNE and its adducts can indicate the severity of diabetic complications. In diabetes, oxidative stress mediated by 4-HNE can cause multiple pathophysiological changes and cause a variety of complications, seriously affecting the quality of life of diabetic patients.

## 3 Pathological Mechanism and Risk Factors of Ferroptosis

In fact, among the many pathological mechanisms of delayed wound healing in diabetic ulcers, the idea that hyperglycemia-induced free radicals and oxidative stress lead to cell death and exacerbate the delayed healing of diabetic ulcers has received extensive attention in recent years. The study of cell death patterns is very important for elucidating the molecular mechanism of disease occurrence. As a newly discovered mode of cell death, ferroptosis has been shown to play a role in many diseases. The following is an introduction to the factors that have a major impact on it.

### 3.1 Reactive Oxygen Species and Ferroptosis

In the electron transport chain, mitochondria produce a large amount of Reactive Oxygen Species (ROS) during normal energy metabolism. These ROS include a series of chemical substances such as superoxide, peroxide and free radicals. ROS are also generated in the following processes, such as the hydroxylation of hypoxanthine to uric acid by xanthine, the catalytic reaction of cytochrome P450, microsomes, NADPH oxidase, cyclooxygenase, uncoupled nitric oxide synthase, lipoxygenase, and peroxisome oxidized fatty acids, etc. In addition, in the process of the body’s fight against infection, activated neutrophils, eosinophils, and macrophages also produce ROS. These signal pathways which are activated by ROS regulate senescence or cell death and have been implicated in cancer, ischemia-reperfusion injury during transplantation, and aging-related neurodegenerative diseases (Nakamura et al., 2019). It can eliminate excess ROS during the reaction catalyzed by antioxidants (enzymes and non-enzymes), superoxide dismutase, glutathione peroxidase, and catalase. When the generation and removal rate of active oxygen is not balanced, it will cause oxidative stress. The resulting excessive manufacture of free radicals may harm DNA, lipids, and proteins (Therond, 2006). Metals which are activated by redox, especially iron ions, can promote the accumulation of ROS in cells through the Fenton reaction. In the Fenton reaction, iron ions catalyze the decomposition of H_2_O_2_ to produce superoxide radicals and superoxide radicals eventually reduce ferric ions to produce ferrous ion and O_2_ (Touati, 2000). Under normal physiological conditions, redox-active ferrous ions are maintained in a low concentration range in the form of unstable iron pools to maintain metabolic needs (Chong et al., 2014), and excess ferrous ions are isolated in proteins, involving ferritin to avoid toxic reactions. Nonetheless, under oxidative stress situations, lofty concentrations of superoxide can lead iron-containing compounds (including heme and ferritin) to release ferrous ions. In the process of ferroptosis, the glutamate/cystine antiporter solute carrier family 7 member 11 (SLC7A11) and the consume of glutathione lead to the accumulation of iron-dependent ROS (Dixon and Stockwell, 2014). In addition, glucose and glutamine metabolism will decrease the levels of glutathione and glutathione peroxidase 4 (GPX4), resulting in the production of iron-independent ROS. Although an essential sign of ferroptosis is that ROS is involved in peroxidation, the others are unclear (Su et al., 2019).

### 3.2 GPX4 Deficiency and Ferroptosis

GPX4 is an antioxidant enzyme that neutralizes lipid peroxides and protects the fluidity of cell membranes (Wu et al., 2019). GPX4 protects cell membrane from peroxidation damage by using glutathione as an auxiliary factor to accelerate the reduction of lipid peroxide (Stockwell et al., 2020). Glutathione reductase and NADPH/H^+^ reduce glutathione disulfide (GSSG), thereby promoting reduced glutathione cycle. The catalytic site is located on the selenocysteine residue of GPX4, and the electrophilic RSL-3 binds to the nucleophilic part of the selenocysteine on the active site of GPX4 to achieve ligand binding (Yang et al., 2016). Therefore, RSL-3 can directly inhibit the activity of GPX4 and induce ferroptosis. Glutathione is a cofactor of GPX4, and the level of glutathione in the cell is also affected by the function of SLC7A11 in the cystine-glutamate antiporter system (Seiler et al., 2008). Erastian, sulfasalazine, and sorafenib can all inhibit the expression of SLC7A11 and cause ferroptosis.

### 3.3 Iron and Ferroptosis

Co-treatment with iron chelator deferoxamine can inhibit ROS accumulation and cell death. Therefore, some studies believe that iron is involved in ferroptosis from the beginning (Dixon et al., 2012). The survey of Dixon et al. (2012) showed that iron regulatory protein 2 (IRP2) regulates the degrees of cellular iron and can inhibit ferroptosis which is induced by erastin. IRP2 binds to iron-responsive elements (IRES). The structure of IRES is a stem loop, and IRES is located at the 5′-UTR and TFRC of ferritin and ferritin, and the 3′-UTR of divalent metal ion transporter 1 (DMT1), inhibiting the ubiquitination of mRNA (Theil, 1990). F-box and leucine-rich repeat protein 5 (FBXL5) is an E3 ubiquitin ligase that regulates cellular and systemic iron homeostasis by mediating iron regulatory protein 2 (IRP2) degradation (Ruiz and Bruick, 2014). However, iron chelation can aim any protein which is iron-involving or relay on iron in the cell, including NADPH oxidase, iron-containing lipoxygenase, xanthine oxidase, etc. The study by Gao et al. (2015) found that transferrin makes an important impact on the induction of ferroptosis, and the importance of iron in the ferroptosis process has been further confirmed. With the approaches of gene silencing, size exclusion grading, and mass spectrometric detection, this study proved that transferrin and glutamine underlied amino acid-induced ferroptosis, especially in the absence of cystine. This regimen is considered the best option for inducing ferroptosis for due to reduced cystine, low levels of glutathione or antioxidant, and iron-loaded transferrin is an ideal substrate and condition for ROS generation in cell culture. Heat shock protein family B member 1 (HSPB1), which reduces intracellular iron concentration, also affects iron sensitivity (Sha et al., 2021). Therefore, the inactivation of HSPB1 is conducive to iron accumulation and erastin-induced ferroptosis. It is worth noting that although elevated iron levels promote ferroptosis, and iron chelation can inhibit this process (Dixon et al., 2012). It has been showed that early L-glutamate can inhibits cystine transport (Glowacka et al., 2002) and leads to nerve cell death. In addition, by inhibiting the hypoxia-inducible factor proline hydroxylase, the oxidative toxicity of glutamate to neurons can also be eliminated by iron chelation (Wlaschek et al., 2019). Takahashi et al. proposed that iron complexing agents can inhibit ferroptosis by inhibiting iron-dependent hypoxia-inducible factor proline hydroxylase. Due to the lack of aconitase activity, IRP2 does not directly sense intracellular iron levels (Tian et al., 2013). In contrast, iron/oxygen receptors are similar to hypoxia-inducible factor prolyl hydroxylase and they are FBXL5 ubiquitin ligases that regulates IRP2 degradation. Studies by LaVaute et al. (2001) and Salvatore et al. (2005) have shown that before neurodegeneration occurs, mice targeted to knock out the IRP2 gene have elevated levels of ferritin and iron in the mouse brain white matter.

### 3.4 Lipid Peroxidation and Ferroptosis

Ferroptosis is mainly caused by a decrease in GPX4 enzyme activity, which leads to a decrease in its ability to remove lipid peroxides. The oxidation of polyunsaturated fatty acids by lipoxygenase can result in the agglomeration of peroxides, which leads to the production of lipid peroxidation decomposition outputs. When polyunsaturated fatty acids stimulate cells to produce RSL-3 to induce ferroptosis, monounsaturated fatty acids like oleic acid may neutralize and protect cells from ferroptosis (Yang et al., 2014). Doll et al. (2017) reported that acyl-CoA synthase long-chain family member 4 (ACSL4) oxidized the accumulation of cell membrane phospholipids to promote ferroptosis. The studies of Kagan et al. (2017) confirmed that oxidized phosphatidylethanolamine is a lipid peroxide produced by ACSL4 and can be used as an inducer of ferroptosis. The researchers demonstrated through the clustered regularly interspaced short palindromic repeats (CRISPR) technology that after the ACSL4 gene was knocked out, the production of lipid peroxides and ferroptosis in cells were inhibited, while the overexpression of ACSL4 can reverse this result. D’Herde and Krysko (2017) identified oxidized phospholipids produced during ferroptosis by the analyze of liquid chromatography tandem-mass spectrometry (LC-MS/MS), which were extracted from RSL-3 sensitive cultured cells. Moreover, *in vivo* and *in vitro* models, only one type of phosphatidylethanolamine phospholipid has been identified as a lipid that induces ferroptosis. Phosphatidylethanolamine containing two fatty acyl groups of ACSL4 activated arachidonic and adrenergic acyl groups has been shown to be the death signal of ferroptosis (Latunde-Dada, 2017). ACSL4 gene knockout can attenuate the effect of arachidonic acid formyl or epinephrine formyl esterification to phosphatidylethanolamine. Essentially, ACSL4 catalyzes the linkage of arachidonic or adrenal to produce arachidonic or adrenal derivatives, these derivatives are then esterified to phosphatidylethanolamine by lysophosphatidylcholine acyltransferase 3 (LPCAT3), then 15-lipoxygenase (15-LOX) oxidizes phosphatidylethanolamine to produce lipid hydrogen peroxide. GPX4 can reduce the accumulation of lipid hydrogen peroxide, thereby inhibiting ferroptosis (Latunde-Dada, 2017). When the antioxidant enzyme GPX4 reduces lipid peroxides, the antioxidant vitamin E (α-Tocopherol) has been shown to regulate ferroptosis by inhibiting lysyl oxidase (LOX) (Zhang et al., 2022). Vitamin E has the ability to scavenge hydroxyl free radicals, and it also competes for substrate binding sites to inhibit LOX (Kagan et al., 2017). Although the esterified vitamin E analogs α-Tocopherol succinate or α-Tocopherol phosphate cannot generate oxygen free radicals, it can inhibit LOX activity by competing for the binding sites of polyunsaturated fatty acid substrates (Lebold and Traber, 2014). Vitamin E has been shown to inhibit cell ferroptosis *in vitro* and in GPX4−/− gene knockout mice (Seiler et al., 2008; Dixon et al., 2012; Wortmann et al., 2013; Friedmann Angeli et al., 2014; Carlson et al., 2016). Extra inhibitors of ferroptosis cover ferostatin-1, liproxstatin-1 along with coenzyme Q. In addition, different cell types have different sensitivity to ferroptosis inducers (Carlson et al., 2016). Some lymphoma cells have defects in the sulfur transport pathway and rely on the source of extracellular cystine and cysteine. For example, when sulfasalazine is co-cultured with cysteine-secreting fibroblasts or 2-based ethanol is added, sulfasalazine-induced ferroptosis in B cell lymphoma cells is inhibited. It can be seen that the inhibition of GPX4, the depletion of glutathione and the increase of lipoxygenase activity promote the accumulation of polyunsaturated fatty acids and the production of fatty acid-free radicals, thereby inducing the occurrence of cell ferroptosis, cause tissue damage, and further aggravate pathological processes including diabetes, periventricular leukomalacia, acute kidney disease, cancer, and other diseases (Otasevic et al., 2021).

## 4 Ferroptosis Inducers and Inhibitors, and Their Effects on Oxidative Stress and Lipid Peroxidation

### 4.1 Ferroptosis Inducers

There are two main types of ferroptosis inducers (Table 2). The first type can act through cystine-glutamate transporters (system X_C_^−^), including erastin and glutamate, while the second type, RSL3 and DP17, can directly inhibit the activity of glutathione peroxidase (GPX) (Yang et al., 2014; Liang et al., 2019).

**TABLE 2:** Ferroptosis inducers and inhibitors.

Reagents	Target	Function	Effect
Erastin	System X_C_^−^	Restrains systemic X_C_^−^ activity, inactivates the GPX4 enzyme, causes GSH depletion and induces ROS formation	Induces ferroptosis
RSL3	GPX4	Binds and inactivates GPX4 and develops lipid ROS levels	Induces ferroptosis
Glutamate	System X_C_^−^	Inhibits cystine uptake and glutathione synthesis at high concentrations	Induces ferroptosis
Artemisinin	Unknown	Produces ROS and causes oxidative stress	Induces ferroptosis
Vitamin E (Alpha-tocopherol)	5-lipoxygenase	Inhibits ferroptosis *via* LOX suppression and controls cellular redox homeostasis	Inhibits ferroptosis
Deferoxamine	Fe^2+^	Chelates excess iron, inhibits ROS accumulation and cell death	Inhibits ferroptosis
Ferrostatin-1	ROS	Decrease the accumulation of lipid ROS by collecting radical antioxidants without inhibition of LOX activity	Inhibits ferroptosis
Liproxstain-1	ROS	Decrease the accumulation of lipid ROS by collecting radical antioxidants without inhibition of LOX activity	Inhibits ferroptosis
Zileuton	5-lipoxygenase	Controls cellular redox homeostasis	Inhibits ferroptosis

Erastin induces cell death in two ways. One is that erastin binds to mitochondrial VDAC2/3 in BJeLR cells and induces ferroptosis with aberrant ROS generated by the mitochondrial oxidative respiratory chain (Yang et al., 2020). Another is that erastin restrains systemic X_C_^−^ activity and causes the depletion of GSH, thereby inactivating the GPX4 enzyme, which then induces ROS formation, selectively inducing ferroptosis in HRASV12 mutant BJeLR cells (Dixon et al., 2012). RSL3 binds and inactivates GPX4, thereby inhibiting the peroxidase activity of GPX4 and developing lipid ROS levels to induce ferroptosis (Ju et al., 2021). Glutamate is an essential molecule that causes ferroptosis, thereby cells cannot initiate ferroptosis without this amino acid. Artemisinin produces ROS and causes oxidative stress in cancer cells, leading to cell death (Ooko et al., 2015). Artemisinin induces iron- and ROS-dependent cell killing in pancreatic ductal adenocarcinoma cell lines, suggesting that Artemisinin can act as a specific inducer of ferroptosis in pancreatic cancer cells (Sui et al., 2018).

### 4.2 Ferroptosis Inhibitors

The targets and functions of ferroptosis inhibitors are shown in Table 2. Ferrostatin-1 and liproxstatin-1 decrease the accumulation of lipid ROS by collecting radical antioxidants without inhibition of LOX activity and therefore exhibit no effects on ferroptosis (Yin et al., 2011; Zilka et al., 2017; Li et al., 2021). The antioxidants vitamin E and α-Tocotrienol inhibit ferroptosis *via* LOX suppression (Kagan et al., 2017; Elakkad et al., 2021). Zileuton, a LOX inhibitor, protects neurocytes from glutamate-induced oxidative damage by inhibiting ferroptosis (Liu et al., 2015).

## 5 Ferroptosis and Diabetic Ulcers

### 5.1 Lack of GPX4

GPX4 is a unique member of the selenoprotein family and is a major scavenger of intracellular lipid peroxides (Sha et al., 2021). Deficiency of selenium in serum or cytoplasm may impair the function of GPX4, eventually leading to the accumulation of lipid peroxides, which in turn leads to ferroptosis (Friedmann Angeli and Conrad, 2018). Possibly due to reduced dietary intake in diabetic patients, selenium is involved in scavenging free radicals and regenerating vitamin E from free radicals (Bolajoko et al., 2017). Significant reductions of selenium, vitamin E, and TAS concentrations were detected in patients with DFU, which may increase the risk of DFU, leading to impaired wound healing (Amini et al., 2021).

### 5.2 Iron Overload

Iron overload has been recognized as a risk factor for organ dysfunction and damage resulting in diseases such as liver and heart disease, diabetes mellitus, and neurodegenerative diseases. Excessive iron levels impair the healing of diabetic ulcers at the molecular and cellular levels (Saberianpour et al., 2021). Iron plays a direct and causal role in the pathogenesis of diabetes mediated by β-Cell exhaustion and insulin resistance (Simcox and McClain, 2013), even in the absence of apparent iron overload (Vari et al., 2007), thus precise control of iron levels in the body is critical for maintaining metabolic homeostasis (Wang et al., 2021). Iron overload can lead to diabetes symptoms such as decreased insulin secretion, metabolic abnormalities, and mitochondrial dysfunction (Huang et al., 2013), and accelerate the development of diabetes with prompting β cell dysfunction (Utzschneider et al., 2014). In addition, Both of patients with diabetes (Ford and Cogswell, 1999) and metabolic syndrome (Vari et al., 2007) have been found higher ferritin levels in serum.

One of the main sources of endothelial oxidative stress and inflammation is hyperglycemia, and iron overload aggravates endothelial dysfunction caused by hyperglycemia (Wu et al., 2020). Iron overload enhances oxidative stress and the NACHT, LRR, and PYD domains-containing protein 3 (NLRP3) inflammasome signaling, triggering the evolution of several inflammatory mediators, resulting in a cascade of inflammatory responses and renal dysfunction in iron-overloaded rats (Chaudhary et al., 2018). However, it can also mediate protective effects such as immune modulation and limiting free radical production. In addition, to control inflammation in critically ill patients, hyperferritinemia is often used clinically as a key acute phase reactant (Kernan and Carcillo, 2017). Hyperferritin, or hyperferritinemia, but is more common in acute phase reactions, as a result of ferritin being released from damaged cells (Beaton and Adams, 2012). For serum ferritin (SF) normal values, most UK laboratories report normal ranges of 300–400 lg/l for adult males and 150–200 lg/l for adult females (Cullis et al., 2018). By definition, transferrin saturation (TS) values greater than 50% in males and greater than 45% in females are defined as elevated. Hyperferritinemia, which uses large amounts of iron-deficient ferritin as an immunomodulator, induces pro-inflammatory cytokines and immunosuppression (Carcillo et al., 2020). And, it can be used as a biomarker of uncontrolled inflammation to measure the effectiveness of interventions.

### 5.3 Ferroptosis Inhibitors

Up to now, there have been many related studies showing that ferroptosis plays an important role in diabetes and its complications, and many ferroptosis inhibitors are closely related to diabetes. With the in-depth study of the pathological mechanism of ferroptosis, many ferroptosis inhibitors have been discovered and identified, such as ferrostatin-1, liproxstatin-1, vitamin E, and Zileuton. In a model of diabetes, ferrostatin-1 reduces accumulation of lipid peroxides and infiltration of macrophage, and increases the number of insulin-associated cells, protecting islets from streptozotocin (STZ)-induced damage (Stancic et al., 2022). Vitamin E is an antioxidant that can reduce plasma glucose concentrations, insulin levels, etc. in diabetic patients (Millen et al., 2004). In addition, vitamin E has also been shown to reduce oxidative stress and oxidative damage in diabetic patients and animal models, and can also protect diabetic patients from oxidative stress and reduce lipid peroxidation (Pazdro and Burgess, 2010).

### 5.4 AGEs

High availability of glucose and/or lipids is characteristic of diabetes and obesity, which is responsible for the increased production of highly reactive carbonyl compounds (Menini et al., 2021). This condition is called “carbonyl stress.” Also known as glycotoxins and lipotoxins, these compounds are characterized by rapid reactions that destroy various molecules in the cell, ultimately forming products called advanced glycation end products (AGEs). AGEs are destructively modified proteins and/or lipids formed under hyperglycemic conditions (Nowotny et al., 2015). It has been extensively reported that AGEs are involved in the pathogenesis of type 2 diabetes and diabetic complications (Nowotny et al., 2015). The Maillard reaction is the most common pathway known to form AGEs. During the Maillard reaction stage, highly reactive carbonyl compounds are formed, including glyoxal, methylglyoxal, or 3-deoxyglucosone (Wells-Knecht et al., 1995; Fu et al., 1996; Mandl-Weber et al., 2001). They are intermediates or by-products of glucose autoxidation, lipid peroxidation or polyol pathways. Increased concentrations of glyoxal, methylglyoxal, and 3-deoxyglucosone were found in the plasma of T2DM patients (Scheijen and Schalkwijk, 2014). AGEs are normally present in the extracellular matrix (ECM), so modified matrix proteins impair the matrix and the interactions between stromal cells (Nowotny et al., 2015). This can lead to cell death, cell differentiation or reduced cell adhesion and migration. Previous studies have shown a potential association between AGEs levels and iron overload (Mirlohi et al., 2018; Chen et al., 2020). In patients with ß-Thalassemia major, iron overload and oxidative stress can increase the formation of AGEs and lead to different complications (Mirlohi et al., 2018). A previous study in diabetic rats found that high doses of ferric iron led to the accumulation of AGEs in the liver. In the testis, high-dose iron increased the AGE-RAGE axis and the expression of AGE uptake receptors such as accessory gene regulator 1 (AGR1) and cluster of differentiation 36 (CD36) (Chen et al., 2020). In a mechanism that interferes with wound healing, accumulation of AGEs leads to the formation of glycosylated collagen and increases oxidative stress (Qing, 2017). Furthermore, AGEs have high affinity to neutrophil accessory gene regulator (AGR) (AGE receptor), inhibiting the transendothelial migration and bactericidal ability of neutrophils (Glowacka et al., 2002). At this time, neutrophils cannot reach the wound site in time, forming an inflammatory zone (Tian et al., 2013; 2016a). Neutrophils bind to AGEs outside vascular tissue and release large amounts of inflammatory cytokines, resulting a longer time of wound healing and the formation of chronic or refractory wounds (Tian et al., 2016b).

### 5.5 HO-1

Heme oxygenase-1 (HO-1), also known as heat shock protein-32 (HSP 32), is an anti-inflammatory, antioxidant and cytoprotective enzyme. HO-1 is a key mediator of ferroptosis and plays an important role in the development of various diseases (Lei et al., 2019). By degrading heme and increasing iron accumulation, excess HO-1 causes iron overload, oxidative stress and lipid peroxidation, ultimately triggering ferroptosis (Chang et al., 2018; Hassannia et al., 2018). In addition to suppressing immune or inflammatory damage, it counteracts oxidative stress caused by hyperglycemia and improves glucose metabolism and insulin sensitization in type 2 diabetes (Ndisang and Jadhav, 2009). HO-1 reduces inflammatory cytokines such as TNF-α and IL-6, enhances the anti-inflammatory and antioxidant functions of diabetic rats, and promotes angiogenesis to accelerate wound healing (Chen et al., 2016). Under hyperglycemic conditions, HO-1 expression was induced and altered in a time-dependent manner. HO-1 expression was elevated to the peak, and decreasing rapidly in the first 24 h. Finally, at the 96th h, it was found that the expression of HO-1 reached at a minimum value, and the oxidative stress index (OSI) of fibroblasts was increased, collagen synthesis of fibroblasts was reduced, proliferation and migration were supressed, and apoptosis was increased (Li et al., 2018). Therefore, delayed wound healing in diabetic mice may be related to delayed HO-1 upregulation, and HO-1 gene transfer can improve wound healing.

## 6 Conclusion and Perspective

Delayed wound healing of diabetic ulcers involves multiple pathological mechanisms, including peripheral nerve and blood vessel injury and increased inflammation cascades. Among various pathogenesis, high glucose-induced lipid peroxidative damage, excessive oxidative stress and ferroptosis are key pathophysiological mechanisms of diabetic ulcers. Ferroptosis features overload of intracellular iron and accumulation of iron-dependent lipid peroxide, and its role in delayed healing of diabetic wounds has received increasing attention. Reducing ferroptosis might suppress inflammation and be beneficial to the generation of pro-angiogenic factors, thereby improving healing of wounds.

Currently, the research of cellular ferroptosis mainly focuses on neurodegeneration, heart disease, kidney disease, liver disease and cancer, while the research on the role of ferroptosis signaling pathway in wound healing disorders of diabetic ulcers is limited. Therefore, further elucidating the effect of cellular ferroptosis on delayed wound healing of diabetic ulcers, and exploring the specific molecular mechanism of ferroptosis-related signaling pathways in the occurrence and development of diabetic ulcers are of great significance for noval clinical therapeutic ideas for diabetic ulcers.

## Glossary

ACSL4: acyl-CoA synthase long-chain family member 4
AGEs: advanced glycation end products
AGR: accessory gene regulator
AGR1: accessory gene regulator 1
Akt3: RAC-gamma serine/threonine-protein kinase
AL-4: angiopoietin-like factor 4
ANGPTL4: angiopoietin-like factor 4
ATF-3: activating transcription factor 3
CD36: cluster of differentiation 36
CRISPR: the clustered regularly interspaced short palindromic repeats
DFU: diabetic foot ulcer
DMT1: divalent metal transporter 1
ECM: extracellular matrix
EGF: epidermal growth factor
eNOS: endothelial nitric oxide synthase
ERK1: extracellular signal-regulated protein kinases 1
FBXL5: F-box and leucine-rich repeat protein 5
GATA2: GATA binding protein 2
GPX: glutathione peroxidase
GPX4: glutathione peroxidase 4
GSSG: glutathione disulfide
HO-1: heme oxygenase-1
HSP 32: heat shock protein-32
HSPB1: heat shock protein family B member 1
IGF-1: insulin-like growth factor-1
IL-6: interleukin 6
IL-10: interleukin-10
iNOS: inducible nitric oxide synthase
IRES: internal ribosomal entry sites
IRP2: iron regulatory protein 2
JAK/STAT3: the Janus kinase-signal transducer and activator of transcription 3
LC-MS/MS: liquid chromatography tandem-mass spectrometry
LOX: lysyl oxidase
LPCTA3: lysophosphatidylcholine acyltransferase 3
MiRNA or MiR: micro RNA
MMP-9: matrix metalloproteinase 9
NADPH: nicotinamide adenine dinucleotide phosphate
NF-kappa B: nuclear factor kappa B
NF-KB19: nuclear transcription factor 19
NLRP3: the NACHT, LRR, and PYD domains-containing protein 3
NRF2: nuclear factor erythroid 2-related factor 2
OSI: oxidative stress index
PDGF: platelet-derived growth factor
PKB: protein kinase B
PUFAs: polyunsaturated fatty acids
RCS: reactive carbonyl species
RIPK1/RIPK3: receptor-interacting protein kinase 1/receptor-interacing protein kinase 3
ROS: reactive oxygen species
SF: ferritin
SLC7A11: the glutamate/cystine antiporter solute carrier family 7 member 11
STZ: streptozotocin
system X_C_^−^: cystine-glutamate transporters
TGF-β: transforming growth factor-beta
TNF-α: necrosis factor alpha
TS: transferrin saturation
VEGF: vascular endothelial growth factor
VEGFR-1: vascular endothelial growth factor receptor 1
VEGFR2: vascular endothelial growth factor receptor 2
4-HNE: 4-Hydroxy-2-nonenal

## References

[B1] Al-Mohaithef M. Abdelmohsen S. A. Algameel M. Abdelwahed A. Y. (2022). Screening for Identification of Patients at High Risk for Diabetes-Related Foot Ulcers: a Cross-Sectional Study. J. Int. Med. Res. 50 (3), 030006052210878. 10.1177/03000605221087815 PMC8966102 35343272

[B2] Alavi A. Sibbald R. G. Mayer D. Goodman L. Botros M. Armstrong D. G. (2014). Diabetic Foot Ulcers. J. Am. Acad. Dermatology 70 (1), e1–18. 10.1016/j.jaad.2013.06.055 24355275

[B3] Amini M. R. Aalaa M. Nasli-Esfahani E. Atlasi R. Sanjari M. Namazi N. (2021). The Effects of Dietary/herbal Supplements and the Serum Levels of Micronutrients on the Healing of Diabetic Foot Ulcers in Animal and Human Models: a Systematic Review. J. Diabetes Metab. Disord. 20 (1), 973–988. 10.1007/s40200-021-00793-4 34178870 PMC8212333

[B4] Arya A. K. Tripathi K. Das P. (2014). Promising Role of ANGPTL4 Gene in Diabetic Wound Healing. Int. J. Low. Extrem. Wounds 13 (1), 58–63. 10.1177/1534734614520704 24659626

[B5] Ayala A. Muñoz M. F. Argüelles S. (2014). Lipid Peroxidation: Production, Metabolism, and Signaling Mechanisms of Malondialdehyde and 4-Hydroxy-2-Nonenal. Oxidative Med. Cell. Longev. 2014, 1–31. 10.1155/2014/360438 PMC4066722 24999379

[B6] Badr G. (2013). Camel Whey Protein Enhances Diabetic Wound Healing in a Streptozotocin-Induced Diabetic Mouse Model: the Critical Role of β-Defensin-1, -2 and -3. Lipids Health Dis. 12, 46–499. 10.1186/1476-511x-12-46 23547923 PMC3622574

[B7] Badr G. Hozzein W. N. Badr B. M. Al Ghamdi A. Saad Eldien H. M. Garraud O. (2016). Bee Venom Accelerates Wound Healing in Diabetic Mice by Suppressing Activating Transcription Factor-3 (ATF-3) and Inducible Nitric Oxide Synthase (iNOS)-Mediated Oxidative Stress and Recruiting Bone Marrow-Derived Endothelial Progenitor Cells. J. Cell. Physiol. 231 (10), 2159–2171. 10.1002/jcp.25328 26825453

[B8] Basu Mallik S. Jayashree B. S. Shenoy R. R. (2018). Epigenetic Modulation of Macrophage Polarization- Perspectives in Diabetic Wounds. J. Diabetes its Complicat. 32 (5), 524–530. 10.1016/j.jdiacomp.2018.01.015 29530315

[B9] Beaton M. D. Adams P. C. (2012). Treatment of Hyperferritinemia. Ann. Hepatology 11 (3), 294–300. 10.1016/s1665-2681(19)30923-8 22481446

[B10] Bhatnagar A. (1995). Electrophysiological Effects of 4-hydroxynonenal, an Aldehydic Product of Lipid Peroxidation, on Isolated Rat Ventricular Myocytes. Circulation Res. 76 (2), 293–304. 10.1161/01.res.76.2.293 7834841

[B11] Bhattacharya S. Aggarwal R. Pal Singh V. Ramachandran S. Datta M. (2015). Downregulation of miRNAs during Delayed Wound Healing in Diabetes: Role of Dicer. Mol. Med. 21 (1), 847–860. 10.2119/molmed.2014.00186 26602065 PMC4818267

[B12] Biswas S. Roy S. Banerjee J. Hussain S.-R. A. Khanna S. Meenakshisundaram G. (2010). Hypoxia Inducible microRNA 210 Attenuates Keratinocyte Proliferation and Impairs Closure in a Murine Model of Ischemic Wounds. Proc. Natl. Acad. Sci. U.S.A. 107 (15), 6976–6981. 10.1073/pnas.1001653107 20308562 PMC2872456

[B13] Bolajoko E. B. Akinosun O. M. Anetor J. Mossanda K. S. (2017). Relationship between Selected Micronutrient Deficiencies and Oxidative Stress Biomarkers in Diabetes Mellitus Patients with Foot Ulcers in Ibadan, Nigeria. Turk J. Med. Sci. 47 (4), 1117–1123. 10.3906/sag-1601-95 29154507

[B14] Brem H. Tomic-Canic M. (2007). Cellular and Molecular Basis of Wound Healing in Diabetes. J. Clin. Invest. 117 (5), 1219–1222. 10.1172/JCI32169 17476353 PMC1857239

[B15] Bryk-Wiązania A. H. Undas A. (2021). Hypofibrinolysis in Type 2 Diabetes and its Clinical Implications: from Mechanisms to Pharmacological Modulation. Cardiovasc Diabetol. 20 (1), 191. 10.1186/s12933-021-01372-w 34551784 PMC8459566

[B16] Capilla-González V. López-Beas J. Escacena N. Aguilera Y. de la Cuesta A. Ruiz-Salmerón R. (2018). PDGF Restores the Defective Phenotype of Adipose-Derived Mesenchymal Stromal Cells from Diabetic Patients. Mol. Ther. 26 (11), 2696–2709. 10.1016/j.ymthe.2018.08.011 30195725 PMC6224797

[B17] Carcillo J. A. Kernan K. K. Horvat C. M. Simon D. W. Aneja R. K. (2020). Why and How Is Hyperferritinemic Sepsis Different from Sepsis without Hyperferritinemia?*. Pediatr. Crit. Care Med. 21 (5), 509–512. 10.1097/pcc.0000000000002285 32358338 PMC8121152

[B18] Carlson B. A. Tobe R. Yefremova E. Tsuji P. A. Hoffmann V. J. Schweizer U. (2016). Glutathione Peroxidase 4 and Vitamin E Cooperatively Prevent Hepatocellular Degeneration. Redox Biol. 9, 22–31. 10.1016/j.redox.2016.05.003 27262435 PMC4900515

[B19] Chan Y. C. Roy S. Khanna S. Sen C. K. (2012). Downregulation of Endothelial microRNA-200b Supports Cutaneous Wound Angiogenesis by Desilencing GATA Binding Protein 2 and Vascular Endothelial Growth Factor Receptor 2. Atvb 32 (6), 1372–1382. 10.1161/ATVBAHA.112.248583 PMC3399424 22499991

[B20] Chang L.-C. Chiang S.-K. Chen S.-E. Yu Y.-L. Chou R.-H. Chang W.-C. (2018). Heme Oxygenase-1 Mediates BAY 11-7085 Induced Ferroptosis. Cancer Lett. 416, 124–137. 10.1016/j.canlet.2017.12.025 29274359

[B21] Chaudhary K. Promsote W. Ananth S. Veeranan-Karmegam R. Tawfik A. Arjunan P. (2018). Iron Overload Accelerates the Progression of Diabetic Retinopathy in Association with Increased Retinal Renin Expression. Sci. Rep. 8 (1), 3025. 10.1038/s41598-018-21276-2 29445185 PMC5813018

[B22] Chen Q.-Y. Wang G.-G. Li W. Jiang Y.-X. Lu X.-H. Zhou P.-P. (2016). Heme Oxygenase-1 Promotes Delayed Wound Healing in Diabetic Rats. J. Diabetes Res. 2016, 1–10. 10.1155/2016/9726503 PMC4699015 26798657

[B23] Chen S.-H. Yuan K.-C. Lee Y.-C. Shih C.-K. Tseng S.-H. Tinkov A. A. (2020). Iron and Advanced Glycation End Products: Emerging Role of Iron in Androgen Deficiency in Obesity. Antioxidants 9 (3), 261. 10.3390/antiox9030261 PMC7139764 32235809

[B24] Cheng T.-L. Chen P.-K. Huang W.-K. Kuo C.-H. Cho C.-F. Wang K.-C. (2018). Plasminogen/thrombomodulin Signaling Enhances VEGF Expression to Promote Cutaneous Wound Healing. J. Mol. Med. 96 (12), 1333–1344. 10.1007/s00109-018-1702-1 30341568

[B25] Chong H. C. Chan J. S. K. Goh C. Q. Gounko N. V. Luo B. Wang X. (2014). Angiopoietin-like 4 Stimulates STAT3-Mediated iNOS Expression and Enhances Angiogenesis to Accelerate Wound Healing in Diabetic Mice. Mol. Ther. 22 (9), 1593–1604. 10.1038/mt.2014.102 24903577 PMC4435481

[B26] Crovetti G. Martinelli G. Issi M. Barone M. Guizzardi M. Campanati B. (2004). Platelet Gel for Healing Cutaneous Chronic Wounds. Transfus. Apher. Sci. 30 (2), 145–151. 10.1016/j.transci.2004.01.004 15062754

[B27] Cullis J. O. Fitzsimons E. J. Griffiths W. J. Tsochatzis E. Thomas D. W. (2018). Investigation and Management of a Raised Serum Ferritin. Br. J. Haematol. 181 (3), 331–340. 10.1111/bjh.15166 29672840

[B28] D'Herde K. Krysko D. V. (2017). Oxidized PEs Trigger Death. Nat. Chem. Biol. 13 (1), 4–5. 10.1038/nchembio.2261 27842067

[B29] Davis F. M. Kimball A. Boniakowski A. Gallagher K. (2018). Dysfunctional Wound Healing in Diabetic Foot Ulcers: New Crossroads. Curr. Diab Rep. 18 (1), 2. 10.1007/s11892-018-0970-z 29362914

[B30] Dham D. Roy B. Gowda A. Pan G. Sridhar A. Zeng X. (2021). 4-Hydroxy-2-nonenal, a Lipid Peroxidation Product, as a Biomarker in Diabetes and its Complications: Challenges and Opportunities. Free Radic. Res. 55 (5), 510–524. 10.1080/10715762.2020.1866756 33336611 PMC8260649

[B31] Dixon D. Edmonds M. (2021). Managing Diabetic Foot Ulcers: Pharmacotherapy for Wound Healing. Drugs 81 (1), 29–56. 10.1007/s40265-020-01415-8 33382445

[B32] Dixon S. J. Lemberg K. M. Lamprecht M. R. Skouta R. Zaitsev E. M. Gleason C. E. (2012). Ferroptosis: an Iron-dependent Form of Nonapoptotic Cell Death. Cell 149 (5), 1060–1072. 10.1016/j.cell.2012.03.042 22632970 PMC3367386

[B33] Dixon S. J. Stockwell B. R. (2014). The Role of Iron and Reactive Oxygen Species in Cell Death. Nat. Chem. Biol. 10 (1), 9–17. 10.1038/nchembio.1416 24346035

[B34] Doll S. Proneth B. Tyurina Y. Y. Panzilius E. Kobayashi S. Ingold I. (2017). ACSL4 Dictates Ferroptosis Sensitivity by Shaping Cellular Lipid Composition. Nat. Chem. Biol. 13 (1), 91–98. 10.1038/nchembio.2239 27842070 PMC5610546

[B35] Dolp R. Rehou S. Pinto R. Trister R. Jeschke M. G. (2019). The Effect of Diabetes on Burn Patients: a Retrospective Cohort Study. Crit. Care 23 (1), 28. 10.1186/s13054-019-2328-6 30691499 PMC6348623

[B36] Elakkad Y. E. Mohamed S. N. S. Abuelezz N. Z. (2021). Potentiating the Cytotoxic Activity of a Novel Simvastatin-Loaded Cubosome against Breast Cancer Cells: Insights on Dual Cell Death via Ferroptosis and Apoptosis. Bctt 13, 675–689. 10.2147/bctt.S336712 PMC8684378 34934357

[B37] Everett E. Mathioudakis N. (2018). Update on Management of Diabetic Foot Ulcers. Ann. N.Y. Acad. Sci. 1411 (1), 153–165. 10.1111/nyas.13569 29377202 PMC5793889

[B38] Farghaly T. A. Al-Hasani W. A. Abdulwahab H. G. (2021). An Updated Patent Review of VEGFR-2 Inhibitors (2017-present). Expert Opin. Ther. Pat. 31 (11), 989–1007. 10.1080/13543776.2021.1935872 34043477

[B39] Ford E. S. Cogswell M. E. (1999). Diabetes and Serum Ferritin Concentration Among U.S. Adults. Diabetes Care 22 (12), 1978–1983. 10.2337/diacare.22.12.1978 10587829

[B40] Friedmann Angeli J. P. Conrad M. (2018). Selenium and GPX4, a Vital Symbiosis. Free Radic. Biol. Med. 127, 153–159. 10.1016/j.freeradbiomed.2018.03.001 29522794

[B41] Friedmann Angeli J. P. Schneider M. Proneth B. Tyurina Y. Y. Tyurin V. A. Hammond V. J. (2014). Inactivation of the Ferroptosis Regulator Gpx4 Triggers Acute Renal Failure in Mice. Nat. Cell Biol. 16 (12), 1180–1191. 10.1038/ncb3064 25402683 PMC4894846

[B42] Fu M.-X. Requena J. R. Jenkins A. J. Lyons T. J. Baynes J. W. Thorpe S. R. (1996). The Advanced Glycation End Product, N∊-(Carboxymethyl)lysine, Is a Product of Both Lipid Peroxidation and Glycoxidation Reactions. J. Biol. Chem. 271 (17), 9982–9986. 10.1074/jbc.271.17.9982 8626637

[B43] Gao M. Monian P. Quadri N. Ramasamy R. Jiang X. (2015). Glutaminolysis and Transferrin Regulate Ferroptosis. Mol. Cell 59 (2), 298–308. 10.1016/j.molcel.2015.06.011 26166707 PMC4506736

[B44] Glowacka E. Banasik M. Lewkowicz P. Tchorzewski H. (2002). The Effect of LPS on Neutrophils from Patients with High Risk of Type 1 Diabetes Mellitus in Relation to IL-8, IL-10 and IL-12 Production and Apoptosis *In Vitro* . Scand. J. Immunol. 55 (2), 210–217. 10.1046/j.1365-3083.2002.01046.x 11896938

[B45] Hajmousa G. Przybyt E. Pfister F. Paredes-Juarez G. A. Moganti K. Busch S. (2018). Human Adipose Tissue-Derived Stromal Cells Act as Functional Pericytes in Mice and Suppress High-Glucose-Induced Proinflammatory Activation of Bovine Retinal Endothelial Cells. Diabetologia 61 (11), 2371–2385. 10.1007/s00125-018-4713-0 30151615 PMC6182662

[B46] Hassannia B. Wiernicki B. Ingold I. Qu F. Van Herck S. Tyurina Y. Y. (2018). Nano-targeted Induction of Dual Ferroptotic Mechanisms Eradicates High-Risk Neuroblastoma. J. Clin. Invest 128 (8), 3341–3355. 10.1172/jci99032 29939160 PMC6063467

[B47] Huang H. Cui W. Qiu W. Zhu M. Zhao R. Zeng D. (2015). Impaired Wound Healing Results from the Dysfunction of the Akt/mTOR Pathway in Diabetic Rats. J. Dermatological Sci. 79 (3), 241–251. 10.1016/j.jdermsci.2015.06.002 26091964

[B48] Huang J. Simcox J. Mitchell T. C. Jones D. Cox J. Luo B. (2013). Iron Regulates Glucose Homeostasis in Liver and muscleviaAMP‐activated Protein Kinase in Mice. FASEB J. 27 (7), 2845–2854. 10.1096/fj.12-216929 23515442 PMC3688748

[B49] Humphries K. M. Yoo Y. Szweda L. I. (1998). Inhibition of NADH-Linked Mitochondrial Respiration by 4-Hydroxy-2-Nonenal. Biochemistry 37 (2), 552–557. 10.1021/bi971958i 9425076

[B50] Icli B. Nabzdyk C. S. Lujan-Hernandez J. Cahill M. Auster M. E. Wara A. K. M. (2016). Regulation of Impaired Angiogenesis in Diabetic Dermal Wound Healing by microRNA-26a. J. Mol. Cell. Cardiol. 91, 151–159. 10.1016/j.yjmcc.2016.01.007 26776318 PMC4764471

[B51] Icli B. Wu W. Ozdemir D. Li H. Cheng H. S. Haemmig S. (2019). MicroRNA-615-5p Regulates Angiogenesis and Tissue Repair by Targeting AKT/eNOS (Protein Kinase B/Endothelial Nitric Oxide Synthase) Signaling in Endothelial Cells. Atvb 39 (7), 1458–1474. 10.1161/ATVBAHA.119.312726 PMC6594892 31092013

[B52] Jee J.-P. Pangeni R. Jha S. K. Byun Y. Park J. W. (2019). Preparation and *In Vivo* Evaluation of a Topical Hydrogel System Incorporating Highly Skin-Permeable Growth Factors, Quercetin, and Oxygen Carriers for Enhanced Diabetic Wound-Healing Therapy. Ijn 14, 5449–5475. 10.2147/IJN.S213883 31409998 PMC6647010

[B53] Jelic M. Mandic A. Maricic S. Srdjenovic B. (2021). Oxidative Stress and its Role in Cancer. J. Can. Res. Ther. 17 (1), 22–28. 10.4103/jcrt.JCRT_862_16 33723127

[B54] Jiang Q.-w. Kaili D. Freeman J. Lei C.-y. Geng B.-c. Tan T. (2019). Diabetes Inhibits Corneal Epithelial Cell Migration and Tight Junction Formation in Mice and Human via Increasing ROS and Impairing Akt Signaling. Acta Pharmacol. Sin. 40 (9), 1205–1211. 10.1038/s41401-019-0223-y 30867543 PMC6786421

[B55] Ju J. Song Y.-n. Wang K. (2021). Mechanism of Ferroptosis: A Potential Target for Cardiovascular Diseases Treatment. Aging Dis. 12 (1), 261–276. 10.14336/AD.2020.0323 33532140 PMC7801281

[B56] Kagan V. E. Mao G. Qu F. Angeli J. P. F. Doll S. Croix C. S. (2017). Oxidized Arachidonic and Adrenic PEs Navigate Cells to Ferroptosis. Nat. Chem. Biol. 13 (1), 81–90. 10.1038/nchembio.2238 27842066 PMC5506843

[B57] Kernan K. F. Carcillo J. A. (2017). Hyperferritinemia and Inflammation. Int. Immunol. 29 (9), 401–409. 10.1093/intimm/dxx031 28541437 PMC5890889

[B58] Kim Y.-M. Youn S.-W. Sudhahar V. Das A. Chandhri R. Cuervo Grajal H. (2018). Redox Regulation of Mitochondrial Fission Protein Drp1 by Protein Disulfide Isomerase Limits Endothelial Senescence. Cell Rep. 23 (12), 3565–3578. 10.1016/j.celrep.2018.05.054 29924999 PMC6324937

[B59] Latunde-Dada G. O. (2017). Ferroptosis: Role of Lipid Peroxidation, Iron and Ferritinophagy. Biochimica Biophysica Acta (BBA) - General Subj. 1861 (8), 1893–1900. 10.1016/j.bbagen.2017.05.019 28552631

[B60] LaVaute T. Smith S. Cooperman S. Iwai K. Land W. Meyron-Holtz E. (2001). Targeted Deletion of the Gene Encoding Iron Regulatory Protein-2 Causes Misregulation of Iron Metabolism and Neurodegenerative Disease in Mice. Nat. Genet. 27 (2), 209–214. 10.1038/84859 11175792

[B61] Leake K. Singhal J. Nagaprashantha L. D. Awasthi S. Singhal S. S. (2012). RLIP76 Regulates PI3K/Akt Signaling and Chemo-Radiotherapy Resistance in Pancreatic Cancer. PLoS One 7 (4), e34582. 10.1371/journal.pone.0034582 22509328 PMC3317991

[B62] Lebold K. M. Traber M. G. (2014). Interactions between α-tocopherol, Polyunsaturated Fatty Acids, and Lipoxygenases during Embryogenesis. Free Radic. Biol. Med. 66, 13–19. 10.1016/j.freeradbiomed.2013.07.039 23920314 PMC3874081

[B63] Lei P. Bai T. Sun Y. (2019). Mechanisms of Ferroptosis and Relations with Regulated Cell Death: A Review. Front. Physiol. 10, 139. 10.3389/fphys.2019.00139 30863316 PMC6399426

[B64] Li M. Wang T. Tian H. Wei G. Zhao L. Shi Y. (2019). Macrophage-derived Exosomes Accelerate Wound Healing through Their Anti-inflammation Effects in a Diabetic Rat Model. Artif. Cells, Nanomedicine, Biotechnol. 47 (1), 3793–3803. 10.1080/21691401.2019.1669617 31556314

[B65] Li Q.-l. Guo R.-m. Zhao K. Lin D.-z. Ye X.-m. Chen L.-h. (2018). Effects of Haem Oxygenase-1 Expression on Oxidative Injury and Biological Behaviours of Rat Dermal Fibroblasts. J. Wound Care 27 (11), 780–789. 10.12968/jowc.2018.27.11.780 30398933

[B66] Li S. Li Y. Wu Z. Wu Z. Fang H. (2021). Diabetic Ferroptosis Plays an Important Role in Triggering on Inflammation in Diabetic Wound. Am. J. Physiology-Endocrinology Metabolism 321 (4), E509–e520. 10.1152/ajpendo.00042.2021 34423682

[B67] Li Z. Guo S. Yao F. Zhang Y. Li T. (2013). Increased Ratio of Serum Matrix Metalloproteinase-9 against TIMP-1 Predicts Poor Wound Healing in Diabetic Foot Ulcers. J. Diabetes its Complicat. 27 (4), 380–382. 10.1016/j.jdiacomp.2012.12.007 23357650

[B68] Liang C. Zhang X. Yang M. Dong X. (2019). Recent Progress in Ferroptosis Inducers for Cancer Therapy. Adv. Mat. 31 (51), 1904197. 10.1002/adma.201904197 31595562

[B69] Liu Y. Wang W. Li Y. Xiao Y. Cheng J. Jia J. (2015). The 5-Lipoxygenase Inhibitor Zileuton Confers Neuroprotection against Glutamate Oxidative Damage by Inhibiting Ferroptosis. Biol. Pharm. Bull. 38 (8), 1234–1239. 10.1248/bpb.b15-00048 26235588

[B70] Long M. Rojo de la Vega M. Wen Q. Bharara M. Jiang T. Zhang R. (2016). An Essential Role of NRF2 in Diabetic Wound Healing. Diabetes 65 (3), 780–793. 10.2337/db15-0564 26718502 PMC4764153

[B71] Mandl-Weber S. Haslinger B. Schalkwijk C. G. Sitter T. (2001). Early Glycated Albumin, but Not Advanced Glycated Albumin, Methylglyoxal, or 3-deoxyglucosone Increases the Expression of PAI-1 in Human Peritoneal Mesothelial Cells. Perit. Dial. Int. 21 (5), 487–494. 11757833

[B72] Melincovici C. S. Boşca A. B. Şuşman S. Mărginean M. Mihu C. Istrate M. (2018). Vascular Endothelial Growth Factor (VEGF) - Key Factor in Normal and Pathological Angiogenesis. Rom. J. Morphol. Embryol. 59 (2), 455–467. 30173249

[B73] Menini S. Iacobini C. Vitale M. Pesce C. Pugliese G. (2021). Diabetes and Pancreatic Cancer-A Dangerous Liaison Relying on Carbonyl Stress. Cancers 13 (2), 313. 10.3390/cancers13020313 33467038 PMC7830544

[B74] Millen A. E. Klein R. Folsom A. R. Stevens J. Palta M. Mares J. A. (2004). Relation between Intake of Vitamins C and E and Risk of Diabetic Retinopathy in the Atherosclerosis Risk in Communities Study. Am. J. Clin. Nutr. 79 (5), 865–873. 10.1093/ajcn/79.5.865 15113727

[B75] Mirlohi M. S. Yaghooti H. Shirali S. Aminasnafi A. Olapour S. (2018). Increased Levels of Advanced Glycation End Products Positively Correlate with Iron Overload and Oxidative Stress Markers in Patients with β-thalassemia Major. Ann. Hematol. 97 (4), 679–684. 10.1007/s00277-017-3223-3 29318368

[B76] Moura J. Børsheim E. Carvalho E. (2014). The Role of MicroRNAs in Diabetic Complications-Special Emphasis on Wound Healing. Genes 5 (4), 926–956. 10.3390/genes5040926 25268390 PMC4276920

[B77] Nakamura T. Naguro I. Ichijo H. (2019). Iron Homeostasis and Iron-Regulated ROS in Cell Death, Senescence and Human Diseases. Biochimica Biophysica Acta (BBA) - General Subj. 1863 (9), 1398–1409. 10.1016/j.bbagen.2019.06.010 31229492

[B78] Narayanan S. Eliasson Angelstig S. Xu C. Grünler J. Zhao A. Zhu W. (2020). HypoxamiR-210 Accelerates Wound Healing in Diabetic Mice by Improving Cellular Metabolism. Commun. Biol. 3 (1), 768. 10.1038/s42003-020-01495-y 33318569 PMC7736285

[B79] Nass N. Vogel K. Hofmann B. Presek P. Silber R.-E. Simm A. (2010). Glycation of PDGF Results in Decreased Biological Activity. Int. J. Biochem. Cell Biol. 42 (5), 749–754. 10.1016/j.biocel.2010.01.012 20083221

[B80] Ndisang J. F. Jadhav A. (2009). Up-regulating the Hemeoxygenase System Enhances Insulin Sensitivity and Improves Glucose Metabolism in Insulin-Resistant Diabetes in Goto-Kakizaki Rats. Endocrinology 150 (6), 2627–2636. 10.1210/en.2008-1370 19228889

[B81] Nowak N. C. Menichella D. M. Miller R. Paller A. S. (2021). Cutaneous Innervation in Impaired Diabetic Wound Healing. Transl. Res. 236, 87–108. 10.1016/j.trsl.2021.05.003 34029747 PMC8380642

[B82] Nowotny K. Jung T. Höhn A. Weber D. Grune T. (2015). Advanced Glycation End Products and Oxidative Stress in Type 2 Diabetes Mellitus. Biomolecules 5 (1), 194–222. 10.3390/biom5010194 25786107 PMC4384119

[B83] Okizaki S.-i. Ito Y. Hosono K. Oba K. Ohkubo H. Amano H. (2015). Suppressed Recruitment of Alternatively Activated Macrophages Reduces TGF-Β1 and Impairs Wound Healing in Streptozotocin-Induced Diabetic Mice. Biomed. Pharmacother. 70, 317–325. 10.1016/j.biopha.2014.10.020 25677561

[B84] Ooko E. Saeed M. E. M. Kadioglu O. Sarvi S. Colak M. Elmasaoudi K. (2015). Artemisinin Derivatives Induce Iron-dependent Cell Death (Ferroptosis) in Tumor Cells. Phytomedicine 22 (11), 1045–1054. 10.1016/j.phymed.2015.08.002 26407947

[B85] Otasevic V. Vucetic M. Grigorov I. Martinovic V. Stancic A. (2021). Ferroptosis in Different Pathological Contexts Seen through the Eyes of Mitochondria. Oxidative Med. Cell. Longev. 2021, 1–16. 10.1155/2021/5537330 PMC8205588 34211625

[B86] Park J. Hwang S. Yoon I.-S. (2017). Advanced Growth Factor Delivery Systems in Wound Management and Skin Regeneration. Molecules 22 (8), 1259. 10.3390/molecules22081259 PMC6152378 28749427

[B87] Pazdro R. Burgess J. R. (2010). The Role of Vitamin E and Oxidative Stress in Diabetes Complications. Mech. Ageing Dev. 131 (4), 276–286. 10.1016/j.mad.2010.03.005 20307566

[B88] Qing C. (2017). The Molecular Biology in Wound Healing & Non-healing Wound. Chin. J. Traumatology 20 (4), 189–193. 10.1016/j.cjtee.2017.06.001 PMC5555286 28712679

[B89] Ruiz J. C. Bruick R. K. (2014). F-Box and Leucine-Rich Repeat Protein 5 (FBXL5): Sensing Intracellular Iron and Oxygen. J. Inorg. Biochem. 133, 73–77. 10.1016/j.jinorgbio.2014.01.015 24508277 PMC3959624

[B90] Saberianpour S. Saeed Modaghegh M. H. Montazer M. Kamyar M. M. Sadeghipour Kerman F. Rahimi H. (2021). Relation between Tissue Iron Content and Polarization of Macrophages in Diabetic Ulcer and the Transitional Zone of Diabetic Ulcers with Major Amputation. Int. J. Low. Extrem. Wounds, 153473462110374. 10.1177/15347346211037448 34402324

[B91] Salvatore M. F. Fisher B. Surgener S. P. Gerhardt G. A. Rouault T. (2005). Neurochemical Investigations of Dopamine Neuronal Systems in Iron-Regulatory Protein 2 (IRP-2) Knockout Mice. Mol. Brain Res. 139 (2), 341–347. 10.1016/j.molbrainres.2005.06.002 16051392

[B92] Scheijen J. L. J. M. Schalkwijk C. G. (2014). Quantification of Glyoxal, Methylglyoxal and 3-deoxyglucosone in Blood and Plasma by Ultra Performance Liquid Chromatography Tandem Mass Spectrometry: Evaluation of Blood Specimen. Clin. Chem. Lab. Med. 52 (1), 85–91. 10.1515/cclm-2012-0878 23492564

[B93] Schürmann C. Goren I. Linke A. Pfeilschifter J. Frank S. (2014). Deregulated Unfolded Protein Response in Chronic Wounds of Diabetic Ob/ob Mice: a Potential Connection to Inflammatory and Angiogenic Disorders in Diabetes-Impaired Wound Healing. Biochem. Biophysical Res. Commun. 446 (1), 195–200. 10.1016/j.bbrc.2014.02.085 24583133

[B94] Seiler A. Schneider M. Förster H. Roth S. Wirth E. K. Culmsee C. (2008). Glutathione Peroxidase 4 Senses and Translates Oxidative Stress into 12/15-lipoxygenase Dependent- and AIF-Mediated Cell Death. Cell Metab. 8 (3), 237–248. 10.1016/j.cmet.2008.07.005 18762024

[B95] Sha W. Hu F. Xi Y. Chu Y. Bu S. (2021). Mechanism of Ferroptosis and its Role in Type 2 Diabetes Mellitus. J. Diabetes Res. 2021, 1–10. 10.1155/2021/9999612 PMC8257355 34258295

[B96] Simcox J. A. McClain D. A. (2013). Iron and Diabetes Risk. Cell Metab. 17 (3), 329–341. 10.1016/j.cmet.2013.02.007 23473030 PMC3648340

[B97] Singh M. Kapoor A. Bhatnagar A. (2015). Oxidative and Reductive Metabolism of Lipid-Peroxidation Derived Carbonyls. Chemico-Biological Interact. 234, 261–273. 10.1016/j.cbi.2014.12.028 PMC4414726 25559856

[B98] Singh M. R. Saraf S. Vyas A. Jain V. Singh D. (2013). Innovative Approaches in Wound Healing: Trajectory and Advances. Artif. Cells, Nanomedicine, Biotechnol., 1–11. 10.3109/10731199.2012.716065 23316788

[B99] Stancic A. Saksida T. Markelic M. Vucetic M. Grigorov I. Martinovic V. (2022). Ferroptosis as a Novel Determinant of β-Cell Death in Diabetic Conditions. Oxidative Med. Cell. Longev. 2022, 1–19. 10.1155/2022/3873420 PMC8938062 35320979

[B100] Stockwell B. R. Jiang X. Gu W. (2020). Emerging Mechanisms and Disease Relevance of Ferroptosis. Trends Cell Biol. 30 (6), 478–490. 10.1016/j.tcb.2020.02.009 32413317 PMC7230071

[B101] Su L.-J. Zhang J.-H. Gomez H. Murugan R. Hong X. Xu D. (2019). Reactive Oxygen Species-Induced Lipid Peroxidation in Apoptosis, Autophagy, and Ferroptosis. Oxidative Med. Cell. Longev. 2019, 1–13. 10.1155/2019/5080843 PMC6815535 31737171

[B102] Sui X. Zhang R. Liu S. Duan T. Zhai L. Zhang M. (2018). RSL3 Drives Ferroptosis through GPX4 Inactivation and ROS Production in Colorectal Cancer. Front. Pharmacol. 9, 1371. 10.3389/fphar.2018.01371 30524291 PMC6262051

[B103] Theil E. C. (1990). Regulation of Ferritin and Transferrin Receptor mRNAs. J. Biol. Chem. 265 (9), 4771–4774. 10.1016/s0021-9258(19)34036-0 2156853

[B104] Therond P. (2006). Dommages créés aux biomolécules (lipides, protéines, ADN) par le stress oxydant. Ann. Pharm. Françaises 64 (6), 383–389. 10.1016/s0003-4509(06)75333-0 17119467

[B105] Tian M. Qing C. Niu Y. Dong J. Cao X. Song F. (2016a). Aminoguanidine Cream Ameliorates Skin Tissue Microenvironment in Diabetic Rats. aoms 1 (1), 179–187. 10.5114/aoms.2016.57595 PMC4754380 26925135

[B106] Tian M. Qing C. Niu Y. Dong J. Cao X. Song F. (2013). Effect of Aminoguanidine Intervention on Neutrophils in Diabetes Inflammatory Cells Wound Healing. Exp. Clin. Endocrinol. Diabetes 121 (10), 635–642. 10.1055/s-0033-1351331 24002897

[B107] Tian M. Qing C. Niu Y. Dong J. Cao X. Song F. (2016b). The Relationship between Inflammation and Impaired Wound Healing in a Diabetic Rat Burn Model. J. Burn Care & Res. 37 (2), e115–e124. 10.1097/bcr.0000000000000171 25407384

[B108] Touati D. (2000). Iron and Oxidative Stress in Bacteria. Archives Biochem. Biophysics 373 (1), 1–6. 10.1006/abbi.1999.1518 10620317

[B109] Utzschneider K. M. Largajolli A. Bertoldo A. Marcovina S. Nelson J. E. Yeh M. M. (2014). Serum Ferritin Is Associated with Non-alcoholic Fatty Liver Disease and Decreased Β-cell Function in Non-diabetic Men and Women. J. Diabetes its Complicat. 28 (2), 177–184. 10.1016/j.jdiacomp.2013.11.007 PMC3943487 24360972

[B110] Vari I. S. Balkau B. Kettaneh A. André P. Tichet J. Fumeron F. (2007). Ferritin and Transferrin Are Associated with Metabolic Syndrome Abnormalities and Their Change over Time in a General Population. Diabetes Care 30 (7), 1795–1801. 10.2337/dc06-2312 17416791

[B111] Wang X. Fang X. Zheng W. Zhou J. Song Z. Xu M. (2021). Genetic Support of a Causal Relationship between Iron Status and Type 2 Diabetes: A Mendelian Randomization Study. J. Clin. Endocrinol. Metab. 106 (11), e4641–e4651. 10.1210/clinem/dgab454 34147035 PMC8530720

[B112] Wells-Knecht K. J. Zyzak D. V. Litchfield J. E. Thorpe S. R. Baynes J. W. (1995). Identification of Glyoxal and Arabinose as Intermediates in the Autoxidative Modification of Proteins by Glucose. Biochemistry 34 (11), 3702–3709. 10.1021/bi00011a027 7893666

[B113] Wlaschek M. Singh K. Sindrilaru A. Crisan D. Scharffetter-Kochanek K. (2019). Iron and Iron-dependent Reactive Oxygen Species in the Regulation of Macrophages and Fibroblasts in Non-healing Chronic Wounds. Free Radic. Biol. Med. 133, 262–275. 10.1016/j.freeradbiomed.2018.09.036 30261274

[B114] Wortmann M. Schneider M. Pircher J. Hellfritsch J. Aichler M. Vegi N. (2013). Combined Deficiency in Glutathione Peroxidase 4 and Vitamin E Causes Multiorgan Thrombus Formation and Early Death in Mice. Circ. Res. 113 (4), 408–417. 10.1161/CIRCRESAHA.113.279984 23770613

[B115] Wu J. Minikes A. M. Gao M. Bian H. Li Y. Stockwell B. R. (2019). Intercellular Interaction Dictates Cancer Cell Ferroptosis via NF2-YAP Signalling. Nature 572 (7769), 402–406. 10.1038/s41586-019-1426-6 31341276 PMC6697195

[B116] Wu W. Yuan J. Shen Y. Yu Y. Chen X. Zhang L. (2020). Iron Overload Is Related to Elevated Blood Glucose Levels in Obese Children and Aggravates High Glucose-Induced Endothelial Cell Dysfunction *In Vitro* . BMJ Open Diab Res. Care 8 (1), e001426. 10.1136/bmjdrc-2020-001426 PMC7368571 32675293

[B117] Yang W. S. Kim K. J. Gaschler M. M. Patel M. Shchepinov M. S. Stockwell B. R. (2016). Peroxidation of Polyunsaturated Fatty Acids by Lipoxygenases Drives Ferroptosis. Proc. Natl. Acad. Sci. U.S.A. 113 (34), E4966–E4975. 10.1073/pnas.1603244113 27506793 PMC5003261

[B118] Yang W. S. SriRamaratnam R. Welsch M. E. Shimada K. Skouta R. Viswanathan V. S. (2014). Regulation of Ferroptotic Cancer Cell Death by GPX4. Cell 156 (1-2), 317–331. 10.1016/j.cell.2013.12.010 24439385 PMC4076414

[B119] Yang Y. Luo M. Zhang K. Zhang J. Gao T. Connell D. O. (2020). Nedd4 Ubiquitylates VDAC2/3 to Suppress Erastin-Induced Ferroptosis in Melanoma. Nat. Commun. 11 (1), 433. 10.1038/s41467-020-14324-x 31974380 PMC6978386

[B120] Yin H. Xu L. Porter N. A. (2011). Free Radical Lipid Peroxidation: Mechanisms and Analysis. Chem. Rev. 111 (10), 5944–5972. 10.1021/cr200084z 21861450

[B121] Yu C. Xiao J.-H. (2021). The Keap1-Nrf2 System: A Mediator between Oxidative Stress and Aging. Oxidative Med. Cell. Longev. 2021, 1–16. 10.1155/2021/6635460 PMC8106771 34012501

[B122] Zahid A. A. Ahmed R. Raza Ur Rehman S. Augustine R. Tariq M. Hasan A. (2019). Nitric Oxide Releasing Chitosan-Poly (Vinyl Alcohol) Hydrogel Promotes Angiogenesis in Chick Embryo Model. Int. J. Biol. Macromol. 136, 901–910. 10.1016/j.ijbiomac.2019.06.136 31229545

[B123] Zeng T. Wang X. Wang W. Feng Q. Lao G. Liang Y. (2019). Endothelial Cell-Derived Small Extracellular Vesicles Suppress Cutaneous Wound Healing through Regulating Fibroblasts Autophagy. Clin. Sci. (Lond) 133 (9). 10.1042/cs20190008 30988132

[B124] Zhang X. Wu S. Guo C. Guo K. Hu Z. Peng J. (2022). Vitamin E Exerts Neuroprotective Effects in Pentylenetetrazole Kindling Epilepsy via Suppression of Ferroptosis. Neurochem. Res. 47 (3), 739–747. 10.1007/s11064-021-03483-y 34779994

[B125] Zhao R. Liang H. Clarke E. Jackson C. Xue M. (2016). Inflammation in Chronic Wounds. Ijms 17 (12), 2085. 10.3390/ijms17122085 PMC5187885 27973441

[B126] Zhou H. Li N. Yuan Y. Jin Y.-G. Guo H. Deng W. (2018). Activating Transcription Factor 3 in Cardiovascular Diseases: a Potential Therapeutic Target. Basic Res. Cardiol. 113 (5), 37. 10.1007/s00395-018-0698-6 30094473

[B127] Zhou K. Ma Y. Brogan M. S. (2015). Chronic and Non-healing Wounds: The Story of Vascular Endothelial Growth Factor. Med. Hypotheses 85 (4), 399–404. 10.1016/j.mehy.2015.06.017 26138626

[B128] Zilka O. Shah R. Li B. Friedmann Angeli J. P. Griesser M. Conrad M. (2017). On the Mechanism of Cytoprotection by Ferrostatin-1 and Liproxstatin-1 and the Role of Lipid Peroxidation in Ferroptotic Cell Death. ACS Cent. Sci. 3 (3), 232–243. 10.1021/acscentsci.7b00028 28386601 PMC5364454

